# Supplementary material for: Chromatin sequesters pioneer transcription factor Sox2 from exerting force on DNA
Source: Nat Commun. 2022 Jul 9;13:3988. doi: 10.1038/s41467-022-31738-x (PMC9271091; doi:10.1038/s41467-022-31738-x)
Supplement: Supplementary file 1 — Supplementary Information [file 41467_2022_31738_MOESM1_ESM.pdf]

Supplementary Table 1. Oligonucleotides used in this work

Sequence Description	Source
DNA substrate used in EMSA (Sox2 binding motif in bold)	This paper
GGCCGCTCTAGAGATATCCCCGAGGGTCGAACCATGATGCCGGATCCC**CTTTGTT** ATGCATCTGCCGAGGCCGCTCAATTGGTCGTAGACAGCTCTAGCACCGCTTAAA CGCACGTACGCGCTGTCCCCCGCGTTTTAACCGCCAAGGGGATTACTCCCTAGTCTCCA GGCACGTGTCACATATATACATCCTGTTCCAGTGCCGGTGTCGCTTGGGTCCCGAGG ATATCAAG	

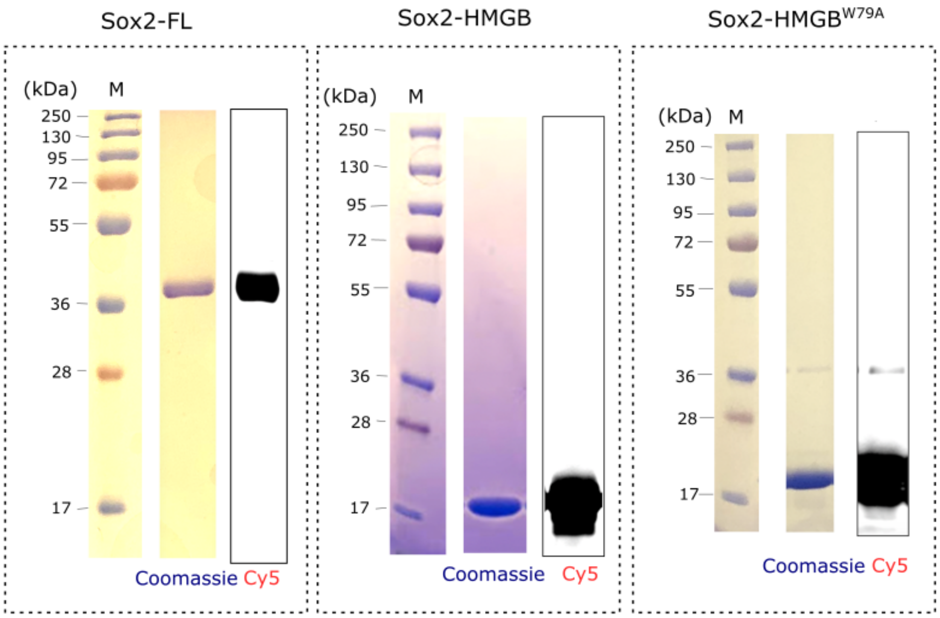

Supplementary Figure 1. Purification and labeling of recombinant human Sox2.
Coomassie stain and Cy5 fluorescence scan of Sox2-FL, Sox2-HMGB, and Sox2-HMGB^{W79A} proteins (among 3 independent preparations).

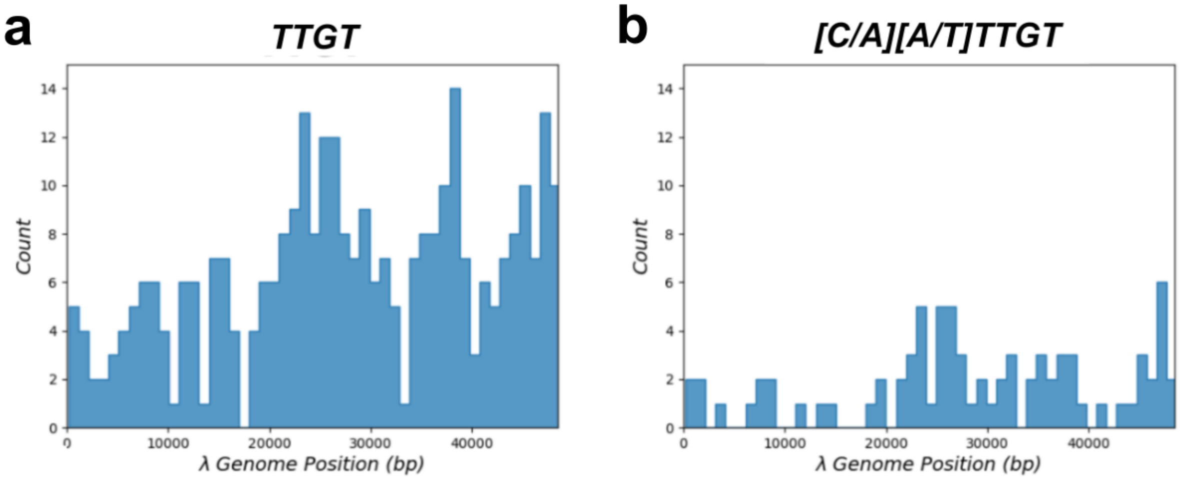

Supplementary Figure 2. Distribution of Sox2 binding motifs on λDNA.

a, Histogram displaying the occurrence of the canonical Sox2 motif TTGT along the λDNA genomic sequence.[1]

b, Histogram displaying the occurrence of the extended Sox2 motif [C/A][A/T]TTGT.[2]

Bin size in the histograms is 1 kb.

Source data are provided as Source Data Supplementary Fig. 2.

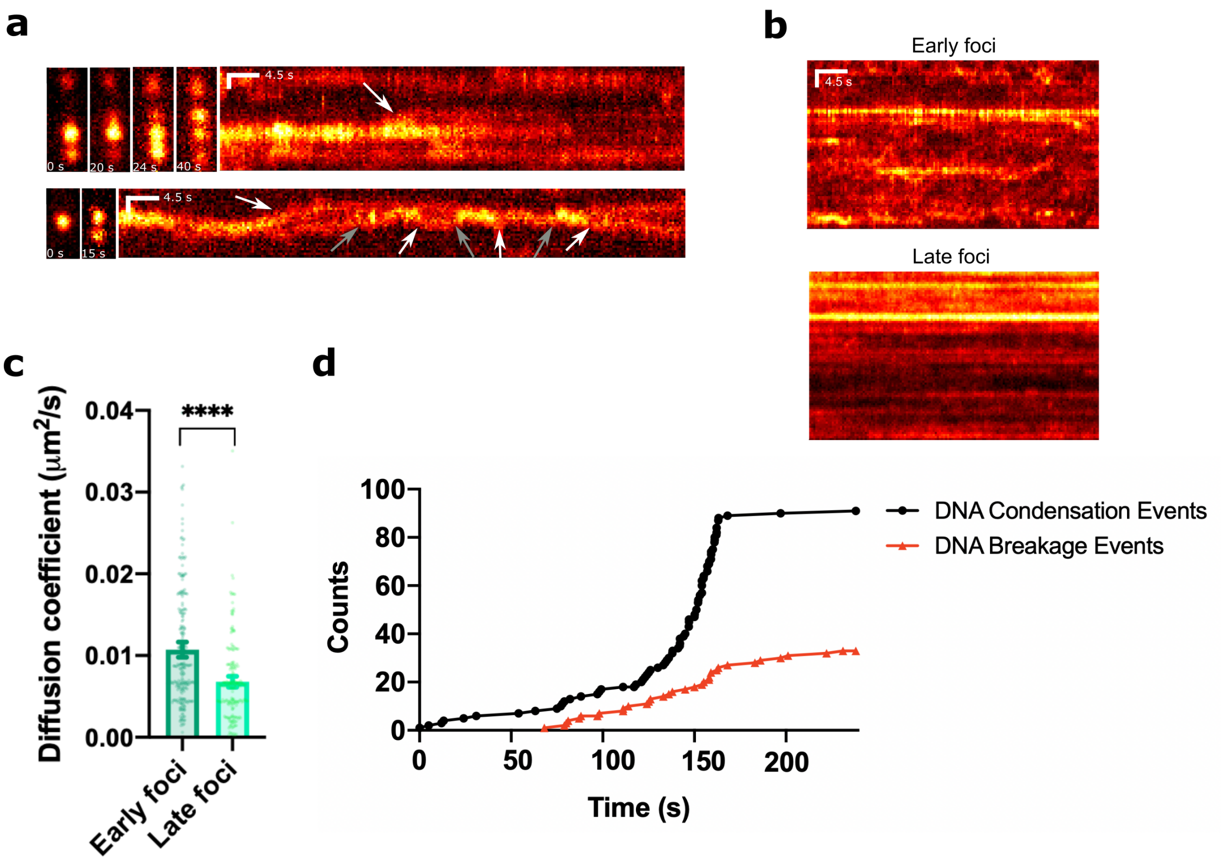

Supplementary Figure 3. Behavior of Sox2 condensates on DNA at different time points.

a, Snapshots and kymographs of two representative double-tethered λDNA molecules displaying fusion (gray arrows) and splitting (white arrows) of Cy5-Sox2 foci. Vertical scale bar denotes 0.5 μm.

b, Kymograph of a representative double-tethered λDNA molecule displaying decreased Sox2 foci mobility over time. Early foci were recorded ~5 min after Sox2 injection, and late foci were recorded ~15 min after injection. Vertical scale bar denotes 1 μm.

c, Bar graph displaying the diffusion coefficient of early Sox2 foci (*n* = 179), recorded at ~5 mins after Sox2 injection, and late Sox2 foci (*n* = 104), recorded at ~15 mins after Sox2 injection, where *n* denotes the number of kymograph traces tracked. Error bars denote 95% CI. Significance was obtained using an unpaired two-sample *t* test (**** $P < 0.0001$).

d, Cumulative incidence of Sox2-mediated DNA condensation and breakage events as a function of time in a representative field of view.

Source data are provided as Source Data Supplementary Fig. 3.

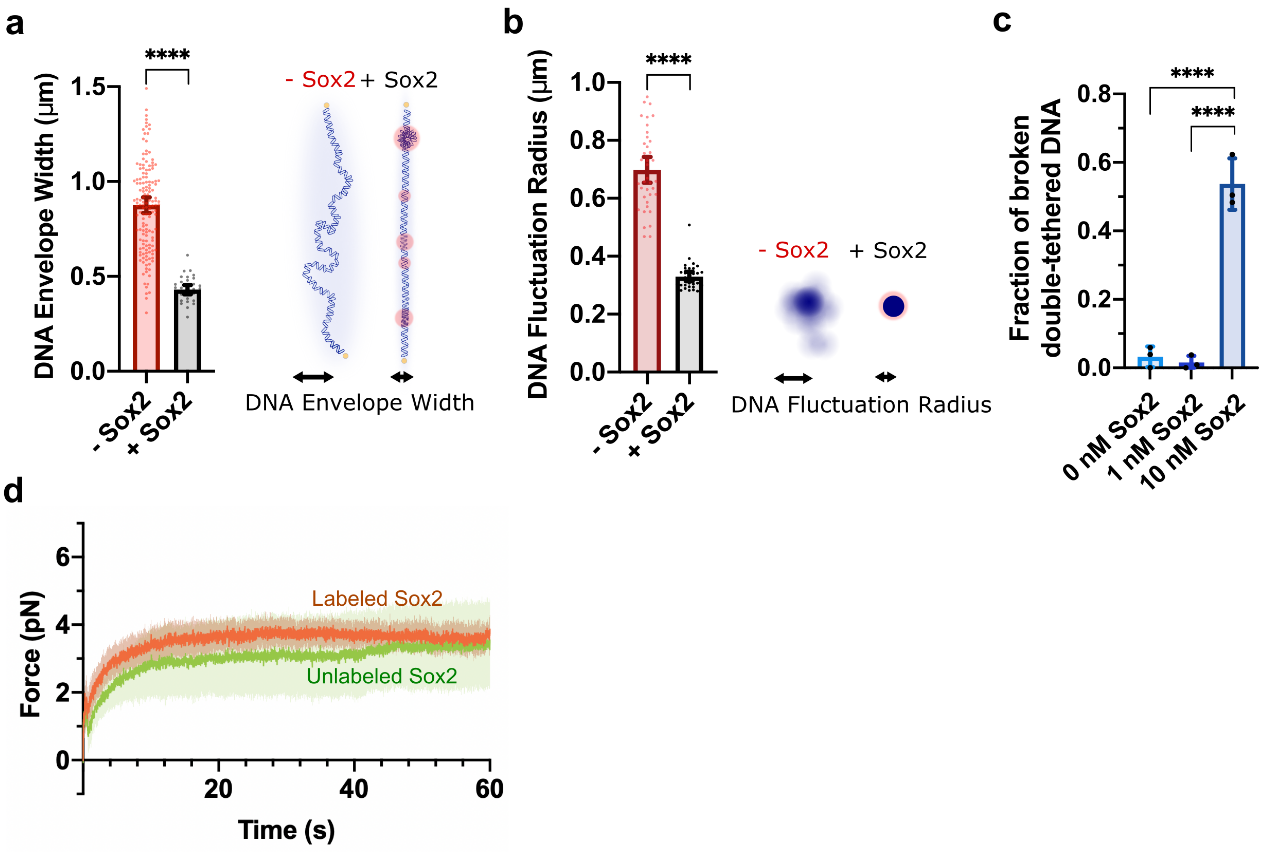

Supplementary Figure 4. Evaluation of the mechanical effects of Sox2:DNA co-condensation using unlabeled Sox2.

a, Bar graph and cartoon showing the DNA envelope width of double-tethered DNA measured in the absence (n = 147) or presence of unlabeled Sox2 (n = 32). Error bars denote 95% CI. Significance was obtained using an unpaired two-sample t test (**** $P < 0.0001$).

b, Bar graph and cartoon showing the fluctuation radius of single-tethered DNA molecules in the absence (n = 38) or presence of unlabeled Sox2 (n = 32). Error bars denote 95% CI. Significance was obtained using an unpaired two-sample t test (**** $P < 0.0001$).

c, Fraction of double-tethered λDNA molecules that broke after 15 min without Sox2 (n = 251), with 1 nM unlabeled Sox2 (n = 131), or with 10 nM unlabeled Sox2 (n = 250). Data are collected from at least three fields of view. Results shown in panels **a-c** are from TIRFM experiments. Error bars denote standard deviation. Significance was obtained using a one-way ANOVA with Dunnett's test for multiple comparisons (**** $P < 0.0001$).

d, Force measurements as a function of time with Cy3-labeled Sox2 (orange, n = 4) or unlabeled Sox2 (green, n = 5) made by the optical tweezers assay. The shades correspond to standard deviation. The Sox2 concentration in these experiments was 100 nM.

Source data are provided as Source Data Supplementary Fig. 4.

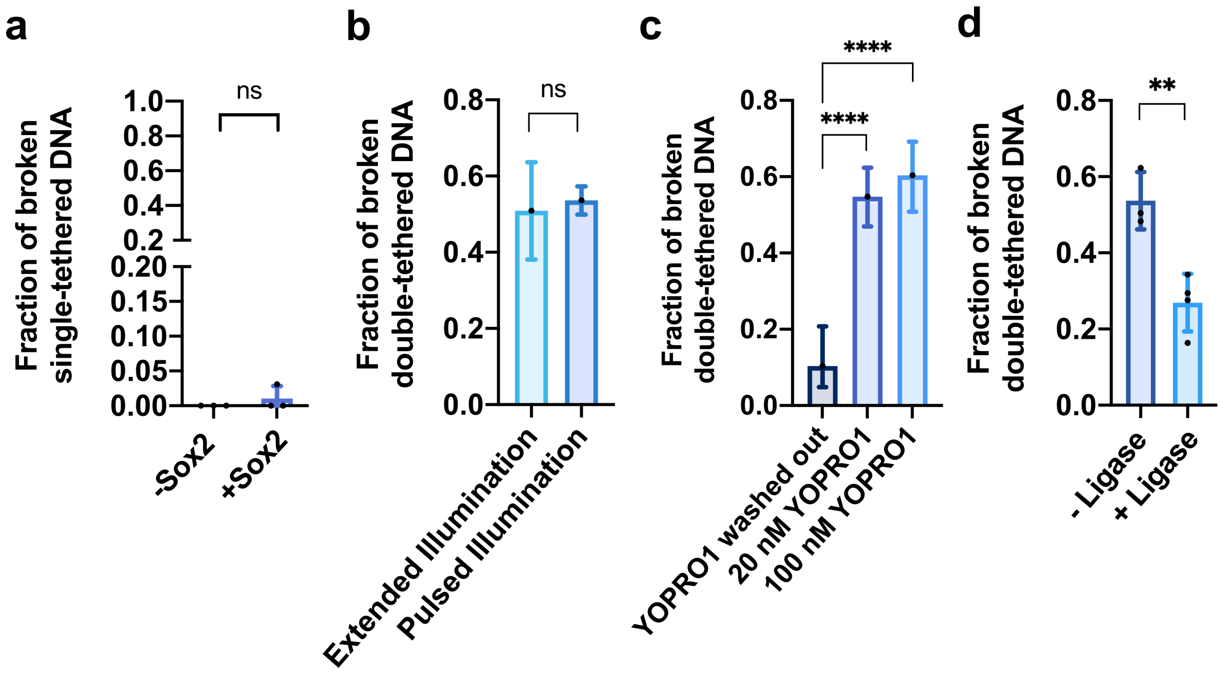

Supplementary Figure 5. Evaluation of Sox2-mediated DNA breakage under different TIRFM experimental conditions.

a, Fraction of single-tethered λDNA molecules that broke after 15 min of imaging in the absence of Sox2 (n = 165) or in the presence of Sox2 (n = 306). Error bars denote standard deviation. Significance was obtained using an unpaired two-sample t test (ns, P = 0.37).

b, Fraction of double-tethered λDNA molecules in the presence of Sox2 that broke after 15 min under different laser illumination schemes. Under “Extended Illumination”, 7.5 min of continuous 488-nm laser illumination was applied (n = 55). Under “Pulsed Illumination”, a single 300-ms pulse of 488-nm laser at the same power was applied (n = 703). Error bars denote standard deviation. Significance was obtained using an unpaired two-sample t test (ns, P = 0.92).

c, Fraction of double-tethered λDNA molecules that broke after 15 min of incubation with Sox2 in the absence of YOPRO1 (washed out with 500 μL of buffer after initial imaging of the DNA) (n = 161), or in the presence of 20 nM YOPRO1 (n = 157) or 100 nM YOPRO1 (n = 106). Error bars denote standard deviation. Significance was obtained using a one-way ANOVA with Dunnett’s test for multiple comparisons (**** P < 0.0001).

d, Fraction of double-tethered λDNA molecules without (n = 250) or with a T4 ligase pre-treatment (n = 184) that broke after 15 min of incubation with Sox2. Error bars denote standard deviation. Significance was obtained using an unpaired two-sample t test (** P = 0.0057). Source data are provided as Source Data Supplementary Fig. 5.

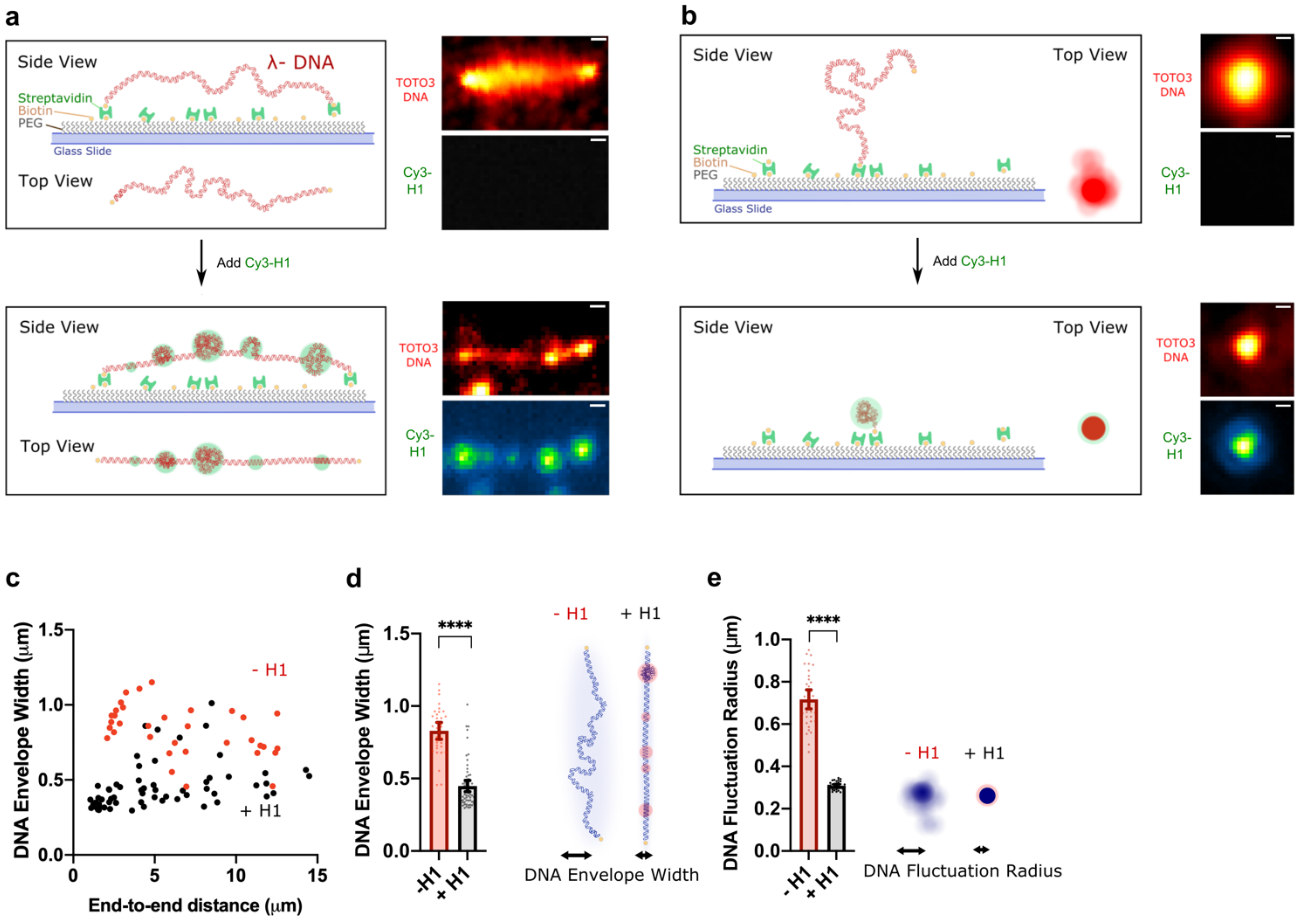

Supplementary Figure 6. DNA condensation by linker histone H1.

a, Schematic (left) and time-averaged projection (right) of a double-tethered λDNA molecule (among 3 independent experiments) stained with 30 nM of the TOTO3 dye and incubated with 150 pM of Cy3-labeled H1. Scale bar, 0.5 μm.

b, Schematic (left) and time-averaged projection (right) of a single-tethered λDNA molecule (among 3 independent experiments) stained with TOTO3 and incubated with Cy3-H1. Scale bar, 0.5 μm.

c, Double-tethered DNA envelope width as a function of the end-to-end distance measured in the absence (n = 35) or presence of H1 (n = 63).

d, Bar graph and cartoon showing a reduction in the average DNA envelope width of double-tethered λDNA molecules in **c** upon H1-mediated DNA condensation. Error bars denote 95% CI. Significance was obtained using an unpaired two-sample t test (**** $P < 0.0001$).

e, Bar graph and cartoon showing the fluctuation radius of single-tethered DNA molecules in the absence (n = 34) or presence of H1 (n = 33). Error bars denote 95% CI. Significance was obtained using an unpaired two-sample t test (**** $P < 0.0001$).

Source data are provided as Source Data Supplementary Fig. 6.

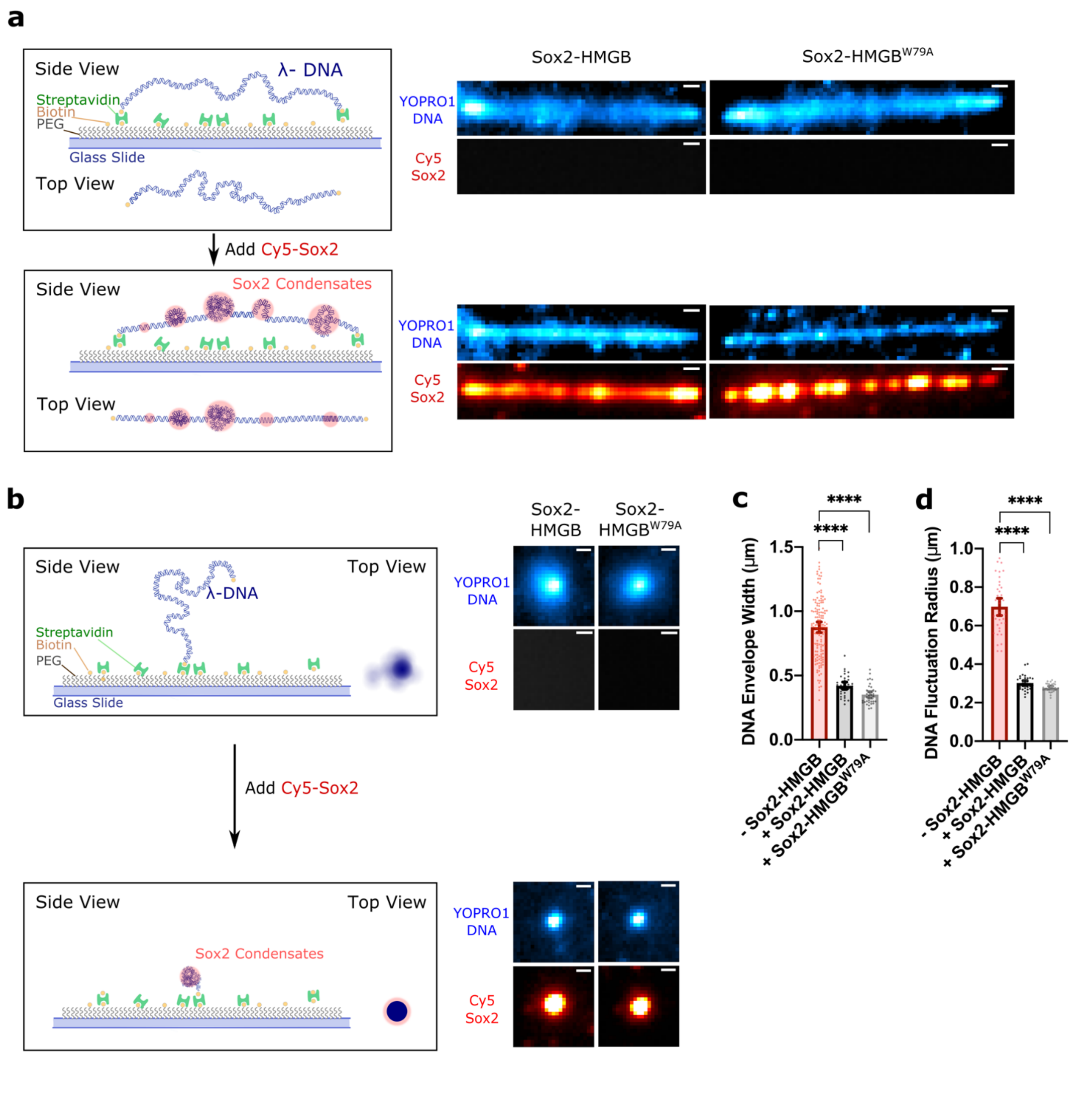

Supplementary Figure 7. DNA condensation mediated by Sox2-HMGB constructs.

a, Schematic and time-averaged projections of double-tethered λDNA stained with YOPRO1 (among 3 independent experiments) and incubated with 10 nM of Cy5-labeled Sox2-HMGB or Sox2-HMGB^{W79A}. Scale bar, 0.5 µm.

b, Schematic and time-averaged projections of single-tethered λDNA stained with YOPRO1 (among 3 independent experiments) and incubated with Cy5-labeled Sox2-HMGB or Sox2-HMGB^{W79A}. Scale bar, 0.5 µm.

c, Bar graph showing the DNA envelope width of double-tethered λDNA molecules without Sox2-HMGB (*n* = 147), or after incubation with Sox2-HMGB (*n* = 34) or with Sox2-HMGB^{W79A} (*n* = 36). Error bars denote 95% CI. Significance was obtained using a one-way ANOVA with Dunnett's test for multiple comparisons (**** $P < 0.0001$).
d, Bar graph showing the fluctuation radius of single-tethered λDNA molecules without Sox2-HMGB (*n* = 38), or after incubation with Sox2-HMGB (*n* = 33) or with Sox2-HMGB^{W79A} (*n* = 32). Error bars denote 95% CI. Significance was obtained using a one-way ANOVA with Dunnett's test for multiple comparisons (**** $P < 0.0001$).
Source data are provided as Source Data Supplementary Fig. 7.

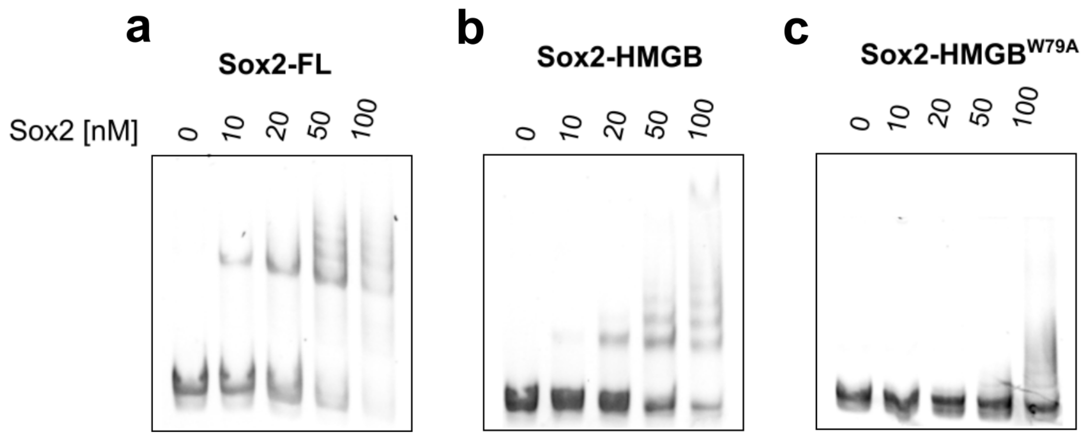

Supplementary Figure 8. EMSA for the binding of different Sox2 constructs to DNA.
a, SYBR-stained gel results for full-length Sox2 (Sox2-FL) binding to a 233-bp DNA that contains a Sox2 binding motif (CTTTGTT).
b, Gel results for Sox2-HMGB binding to the same DNA substrate as in **a**.
c, Gel results for Sox2-HMGB^{W79A} binding to the same DNA substrate as in **a**.

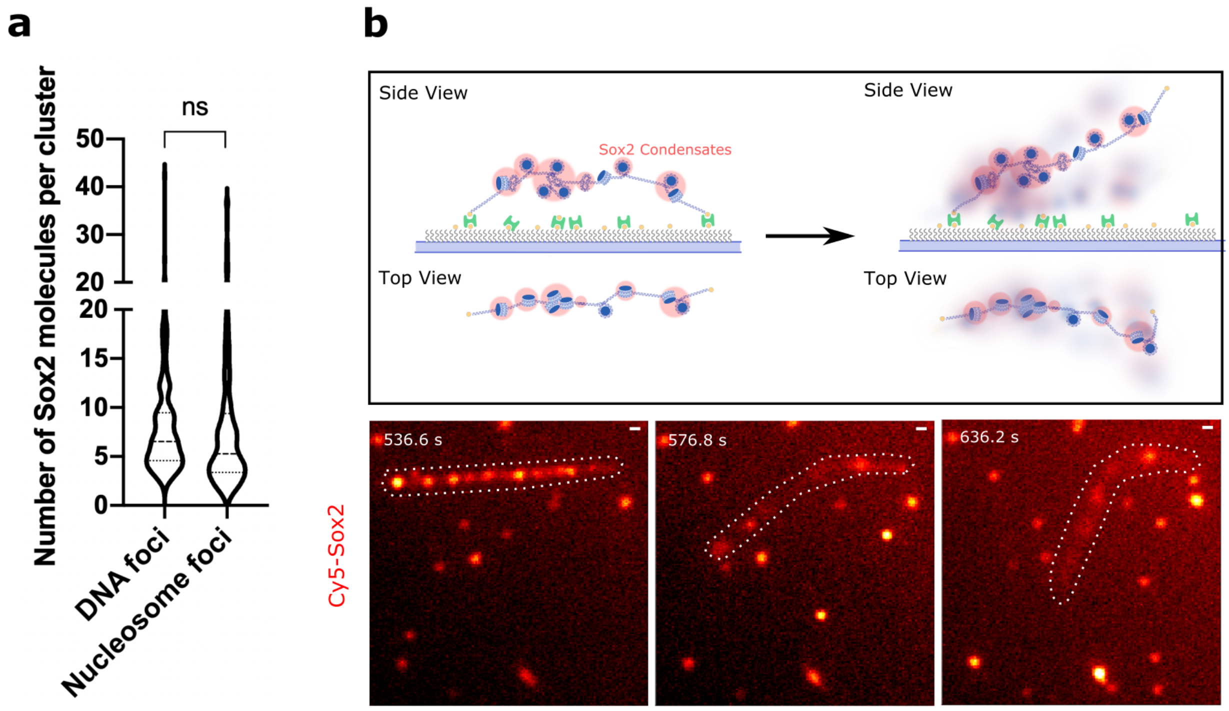

Supplementary Figure 9. Sox2 binding and condensation on nucleosomal DNA.
a, Violin plot showing the distribution of the number of Sox2 molecules within bare DNA foci (n = 167) and the distribution within nucleosome foci (n = 150), where n represents the number of foci analyzed. Significance was obtained using an unpaired two-sample t test (ns, P = 0.96).
b, Schematic (top) and snapshots (bottom) showing Sox2 condensates on a double-tethered nucleosomal DNA molecule. The nucleosomal DNA contour (dashed line) remained in an extended configuration and, when detaching from one anchored end, underwent rigid-body-like fluctuations. Scale bar, 0.5 µm. See also Supplementary Movie 5.
Source data are provided as Source Data Supplementary Fig. 9.

References

1. Schaefer, T. & Lengerke, C. SOX2 protein biochemistry in stemness , reprogramming , and cancer : the PI3K / AKT / SOX2 axis and beyond. *Oncogene* **39**, 278–292 (2020).
2. Hou, L., Srivastava, Y. & Jauch, R. Molecular basis for the genome engagement by Sox proteins. *Semin. Cell Dev. Biol.* **63**, 2–12 (2017).
