# Supplementary material for: Chromatin sequesters pioneer transcription factor Sox2 from exerting force on DNA
Source: Nat Commun. 2022 Jul 9;13:3988. doi: 10.1038/s41467-022-31738-x (PMC9271091; doi:10.1038/s41467-022-31738-x)
Supplement: Supplementary file 3 — Description of Additional Supplementary File [file 41467_2022_31738_MOESM3_ESM.pdf]

Supplementary Movie 1. An example showing Sox2-mediated suppression of single-tethered λDNA fluctuations. The DNA was stained with YOPRO1 (top) and Sox2 was labeled with Cy5 (bottom). The reduction of DNA fluctuation coincided with the increase of Sox2 signal (~ 17 sec). Scale bar: 0.5 μm.

Supplementary Movie 2. An example showing that the formation of Sox2 condensates on double-tethered λDNA causes a reduction in transverse tether fluctuations and eventually DNA breakage. The DNA was stained with YOPRO1 (top) and Sox2 was labeled with Cy5 (bottom). Scale bar: 0.5 μm.

Supplementary Movie 3. An example showing that Sox2 condensate formation mediates the joining of nearby DNA strands. The DNA was stained with YOPRO1 (top) and Sox2 was labeled with Cy5 (bottom). Scale bar: 0.5 μm.

Supplementary Movie 4. An example demonstrating the mechanical effects of Sox2-mediated DNA condensation. Multiple DNA breaking and joining events were observed. The DNA was stained with YOPRO1 for fluorescence visualization. See Fig. 2D for snapshots and cartoon illustrations.

Supplementary Movie 5. An example showing the rigid-body fluctuations of nucleosomal DNA (visualized by the fluorescence signal from bound Cy5- Sox2) after one end detached from the surface. See Supplementary Fig. 9B for snapshots and cartoon illustrations. Scale bar: 0.5 μm.

Supplementary Movie 6. An example showing the fluctuations of singletethered nucleosomal λDNA that persist in the presence of Sox2 (left: without Sox2; right: with Sox2, the same molecule). The nucleosomal DNA was visualized by the fluorescence signal from Cy3-H2B (top) and Sox2 was labeled with Cy5 (bottom). Scale bar: 0.5 μm.
