# Supplementary material for: Chromatin sequesters pioneer transcription factor Sox2 from exerting force on DNA
Source: Nat Commun. 2022 Jul 9;13:3988. doi: 10.1038/s41467-022-31738-x (PMC9271091; doi:10.1038/s41467-022-31738-x)
Supplement: Supplementary file 5 — Reporting Summary [file 41467_2022_31738_MOESM5_ESM.pdf]

nature portfolio

Corresponding author(s): Dr. Shixin Liu

Last updated by author(s): Jun 16, 2022

Reporting Summary

Nature Portfolio wishes to improve the reproducibility of the work that we publish. This form provides structure for consistency and transparency in reporting. For further information on Nature Portfolio policies, see our Editorial Policies and the Editorial Policy Checklist.

Statistics

For all statistical analyses, confirm that the following items are present in the figure legend, table legend, main text, or Methods section.

n/a	Confirmed	
☐	☒	The exact sample size (*n*) for each experimental group/condition, given as a discrete number and unit of measurement
☐	☒	A statement on whether measurements were taken from distinct samples or whether the same sample was measured repeatedly
☐	☒	The statistical test(s) used AND whether they are one- or two-sided<br>*Only common tests should be described solely by name; describe more complex techniques in the Methods section.*
☒	☐	A description of all covariates tested
☒	☐	A description of any assumptions or corrections, such as tests of normality and adjustment for multiple comparisons
☐	☒	A full description of the statistical parameters including central tendency (e.g. means) or other basic estimates (e.g. regression coefficient) AND variation (e.g. standard deviation) or associated estimates of uncertainty (e.g. confidence intervals)
☐	☒	For null hypothesis testing, the test statistic (e.g. *F*, *t*, *r*) with confidence intervals, effect sizes, degrees of freedom and *P* value noted<br>*Give P values as exact values whenever suitable.*
☒	☐	For Bayesian analysis, information on the choice of priors and Markov chain Monte Carlo settings
☒	☐	For hierarchical and complex designs, identification of the appropriate level for tests and full reporting of outcomes
☐	☒	Estimates of effect sizes (e.g. Cohen's *d*, Pearson's *r*), indicating how they were calculated

Our web collection on statistics for biologists contains articles on many of the points above.

Software and code

Policy information about availability of computer code

Data collection	For TIRFM data collection, Metamorph v7.8 software was used. For optical trap data collection, LUMICKs Bluelake v2.1.5 software was used to visualize and process data.
Data analysis	For TIRFM data analysis, Fiji (ImageJ) 2.1.0/1.53c was used. Custom-written scripts were written to further process movies for further downstream analysis, which are available upon request. Kymographs were collected from Fiji (ImageJ) from kymographBuilder plugin and further extracted using Kymotracker 'greedy' tracking algorithm. Mean squared displacement was calculated using a custom python script in MATLAB (R2019a), which is available upon request. For correlation analysis, JaCoP plugin in Fiji was used. PRISM 9 was used to generate graphs in the figures. Details of data analysis were further described in the Methods section of the manuscript.<br>For C-trap data analysis, all raw data generated from experiments were processed and analyzed using a custom GUI Python script titled “C-Trap.h5 File Visualization GUI” (https://harbor.lumicks.com/single-script/c5b103a4-0804-4b06-95d3-20a08d65768f). All specified scripts used to analyze C-trap data can be accessed on Lumicks Harbor site (https://harbor.lumicks.com).

For manuscripts utilizing custom algorithms or software that are central to the research but not yet described in published literature, software must be made available to editors and reviewers. We strongly encourage code deposition in a community repository (e.g. GitHub). See the Nature Portfolio guidelines for submitting code & software for further information.

Data

Policy information about availability of data

All manuscripts must include a data availability statement. This statement should provide the following information, where applicable:

- Accession codes, unique identifiers, or web links for publicly available datasets
- A description of any restrictions on data availability
- For clinical datasets or third party data, please ensure that the statement adheres to our policy

Statistical source data are provided with this paper. Other data are available upon reasonable request.

Field-specific reporting

Please select the one below that is the best fit for your research. If you are not sure, read the appropriate sections before making your selection.

- [x] Life sciences
- [ ] Behavioural & social sciences
- [ ] Ecological, evolutionary & environmental sciences

For a reference copy of the document with all sections, see nature.com/documents/nr-reporting-summary-flat.pdf

Life sciences study design

All studies must disclose on these points even when the disclosure is negative.

Sample size	Sample size was chosen as the number of immobilized lambda DNA molecules visualized in a field of view within each experimental replicate. This was determined by ensuring that we had enough examples to sample the the end-to-end lambda distances from 1 micron to 16 micron.
Data exclusions	For TIRFM experiment, DNA strands were excluded if they were originally adhered to the coverslips, which can discerned via visual inspections of their morphological features. For single-molecule optical tweezer experiments, tethers that display abnormal force-extension behaviors were excluded.
Replication	Reproducibility was ensured by repeating our experiments over the course of multiple days and using preparations of Sox2 from different cultures. We ensured that the behavior of Sox2 proteins on DNA is recapitulated in unlabeled Sox2 as a control. Additionally, we generated Sox2 mutants as well as linker histone H1.4 to make sense of our findings with wild-type Sox2. All attempts at replication were successful. There were at least 3 independent replicate experiments for each study.
Randomization	This was not relevant as there was not grouping used in our study.
Blinding	This was not relevant for our study because the results are not subjective examinations by the the experimenter.

Reporting for specific materials, systems and methods

We require information from authors about some types of materials, experimental systems and methods used in many studies. Here, indicate whether each material, system or method listed is relevant to your study. If you are not sure if a list item applies to your research, read the appropriate section before selecting a response.

Materials & experimental systems

n/a	Involved in the study	
x		Antibodies
x		Eukaryotic cell lines
x		Palaeontology and archaeology
x		Animals and other organisms
x		Human research participants
x		Clinical data
x		Dual use research of concern

Methods

n/a	Involved in the study	
x		ChIP-seq
x		Flow cytometry
x		MRI-based neuroimaging
